# Graviola inhibits hypoxia-induced NADPH oxidase activity in prostate cancer cells reducing their proliferation and clonogenicity

**DOI:** 10.1038/srep23135

**Published:** 2016-03-16

**Authors:** Gagan Deep, Rahul Kumar, Anil K. Jain, Deepanshi Dhar, Gati K. Panigrahi, Anowar Hussain, Chapla Agarwal, Tamam El-Elimat, Vincent P. Sica, Nicholas H. Oberlies, Rajesh Agarwal

**Affiliations:** 1 Department of Pharmaceutical Sciences, Skaggs School of Pharmacy and Pharmaceutical Sciences, University of Colorado Denver, 12850 E. Montview Blvd, C238, Aurora, CO 80045, USA.; 2 University of Colorado Cancer Center, University of Colorado Denver, Aurora, Colorado, USA; 3 Department of Molecular Biology and Biotechnology, Tezpur University, Tezpur, Assam, India; 4 Department of Chemistry and Biochemistry, University of North Carolina at Greensboro, Greensboro, North Carolina, USA

## Abstract

Prostate cancer (PCa) is the leading malignancy among men. Importantly, this disease is mostly diagnosed at early stages offering a unique chemoprevention opportunity. Therefore, there is an urgent need to identify and target signaling molecules with higher expression/activity in prostate tumors and play critical role in PCa growth and progression. Here we report that NADPH oxidase (NOX) expression is directly associated with PCa progression in TRAMP mice, suggesting NOX as a potential chemoprevention target in controlling PCa. Accordingly, we assessed whether NOX activity in PCa cells could be inhibited by Graviola pulp extract (GPE) that contains unique acetogenins with strong anti-cancer effects. GPE (1–5 μg/ml) treatment strongly inhibited the hypoxia-induced NOX activity in PCa cells (LNCaP, 22Rv1 and PC3) associated with a decrease in the expression of NOX catalytic and regulatory sub-units (NOX1, NOX2 and p47^phox^). Furthermore, GPE-mediated NOX inhibition was associated with a strong decrease in nuclear HIF-1α levels as well as reduction in the proliferative and clonogenic potential of PCa cells. More importantly, GPE treatment neither inhibited NOX activity nor showed any cytotoxicity against non-neoplastic prostate epithelial PWR-1E cells. Overall, these results suggest that GPE could be useful in the prevention of PCa progression via inhibiting NOX activity.

Prostate cancer (PCa) is the most common non-cutaneous cancer in men, and according to the American Cancer Society reports, 220,800 new cases and 27,540 deaths from PCa were estimated in the United States in 2015^1^. Fortunately, this disease is mostly diagnosed when it is still localized in the prostate and surrounding organs, and usually it takes a decade or more for the disease to progress to advanced adenocarcinoma and metastatic stages. This long latency-period offers a unique window of opportunity to prevent, inhibit and/or reverse the PCa growth and progression. Recent studies have suggested that one critical element in PCa growth and progression is the aberrant activation of NADPH oxidase (NOX)^2^,^3^,^4^,^5^,^6^,^7^,^8^. The NOX system consists of catalytic subunits (gp91^phox^ [NOX1-5] and p22^phox^) on the membrane and regulatory subunits (p47^phox^, p67^phox^, p40^phox^ and Rac1/2) towards the cytoplasm side. The NOX system passes an electron to oxygen and generates superoxide which is rapidly converted to hydrogen peroxide and causes oxidation of redox-sensitive cysteine residues in target molecules. NOX-generated reactive oxygen species (ROS) act as a secondary messenger and is involved in the regulation of several mitogenic and survival signaling pathways^2^,^3^,^4^,^8^. Importantly, dysregulated NOX-dependent ROS generation is associated with several chronic diseases, including cancer, hypertension, atherosclerosis, diabetic nephropathy, and Alzheimer’s^9^,^10^. For example, NOX-mediated ROS generation activates several oncogenes (e.g. receptor tyrosine kinases, Src, and Ras) as well as inactivates several tumor suppressor genes (e.g. PTEN, p53, TSC2)^7^, and is considered critical in cell transformation as well as an essential element in the maintenance of malignant and resistant phenotype in cancer cells^2^,^3^,^5^,^7^,^10^,^11^,^12^. In this regard, NOX expression is specifically increased in PCa cells, while it is absent or only transiently expressed in normal noncancerous cells; and NOX inhibition (by using chemical inhibitors or small interfering RNAs) retards PCa cells proliferation, invasiveness, androgen independence and angiogenesis, and induces apoptotic death via compromising multiple mitogenic/survival signaling pathways^2^,^3^,^4^,^5^,^7^,^8^,^10^,^13^,^14^,^15^.

Several genetic and epigenetic factors (e.g. oncogenic Ras, hypoxia, IFN-γ, 25-dihydroxy vitamin D3, Toll-like receptor ligand) are known to activate the NOX system^10^,^16^,^17^. Among these factors, hypoxia (low oxygen condition) is one of the key elements that activates NOX, and in turn, NOX stabilizes and activates HIF-1α in a feed-forward mechanism^7^,^10^,^16^,^17^,^18^,^19^. In response to intermittent hypoxia, HIF1α increases the NOX2 expression in the central and peripheral nervous system of mice as well as in cultured cells^17^. Chromatin immunoprecipitation (CHIP) has identified HIF-1α binding to NOX4 gene, and NOX4 induction contributes to maintain ROS levels under hypoxia and supports proliferation of pulmonary artery smooth-muscle cells^16^. Block *et al.* have reported that NOX oxidase (1 and 4) and p22^phox^ regulate HIF-2α expression in VHL-deficient renal cell carcinoma^18^. Others and we have also reported that hypoxia determines PCa aggressiveness, and is an independent prognostic indicator of poor outcomes in PCa^20^,^21^,^22^,^23^. Together, this background provides a strong rationale to target hypoxia-induced NOX activity to prevent and treat PCa.

Graviola (*Annona muricata* L, Family: Annonaceae; popular common names: soursop, custard apple, guanabana) is an evergreen tree widely grown and consumed around the world^24^. Graviola pulp is consumed as juice, in smoothies and for the flavoring of ice cream. Graviola has also long been used in traditional medicine systems for benefits against bacterial and fungal infections, fever, digestive problems, high blood pressure, etc. In two earlier published studies, Graviola fruit powder and leaf/stem powder have shown *in vitro* and *in vivo* anti-cancer efficacy against breast and pancreatic cancer cells, respectively^25^,^26^; however, this is the first study to test Graviola pulp extract (GPE) for its NOX inhibitory efficacy. We rationalized the testing of GPE, as Graviola fruit pulp is the part consumed by humans for centuries. Moreover, for future applications it will be practical and straight forward to translate its beneficial effects into clinic. Results showed a strong inhibitory effect of GPE on NOX activity in PCa cells, reducing their proliferation and clonogenic potential, but without any cytotoxicity towards non-neoplastic prostate epithelial cells.

## Results

### NOX1 and p67^phox^ expression is associated with disease stage and aggressiveness in TRAMP (Transgenic adenocarcinoma of the mouse prostate) prostate tissue

**Figure 1:**
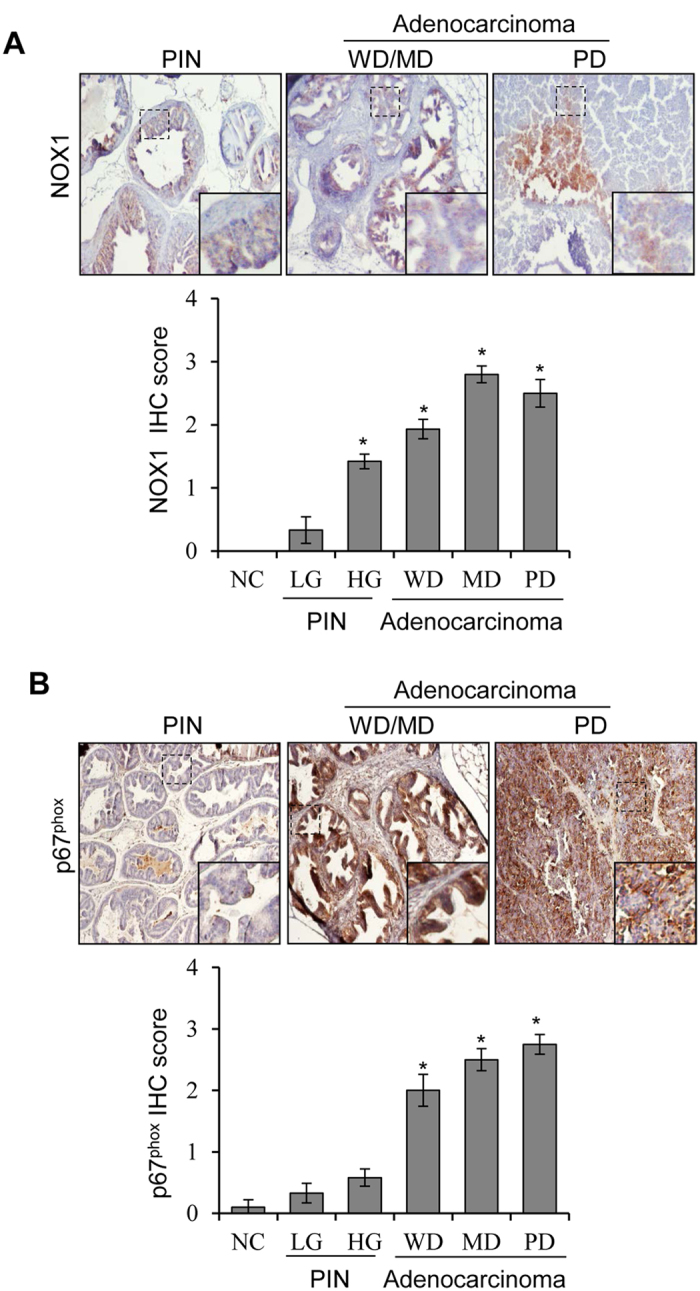
NOX expression is associated with disease progression in TRAMP mice. **(A,B)** NOX1 and p67^phox^ expression was analyzed by IHC in TRAMP prostate tissue with different stages of the disease as well as in non-transgenic mice prostate tissue as negative control. Representative photographs are presented at 100x; inset represent further magnification of a part of the photograph. Immunoreactivity (represented by brown staining) of NOX1 and p67^phox^ was scored as described in methods. Abbreviations: NC: Negative control; LG: Low-grade; HG: High-grade; PIN: Prostate intra-epithelial neoplasia; WD: Well differentiated; MD: Moderately differentiated; PD: Poorly differentiated. *p ≤ 0.001.

TRAMP mice spontaneously exhibit different stages of PCa including low-grade prostate intra-epithelial neoplasia (LGPIN), high-grade PIN (HGPIN), and adenocarcinoma [well-differentiated (WD), moderately differentiated (MD) and poorly differentiated (PD)]^27^,^28^. We first analyzed the NOX1 and p67^phox^ expression in different stages of the disease. Results showed that both NOX1 and p67^phox^ expression are either absent or extremely low in the normal prostate tissue in non-transgenic mice, but their expression increased with the disease progression from PIN to adenocarcinoma in TRAMP prostate tissue (Fig. 1A,B). These results suggested that NOX could be an important target to prevent PCa progression.

### Comparison of various parts of Graviola for cytotoxicity against PCa cells

**Figure 2:**
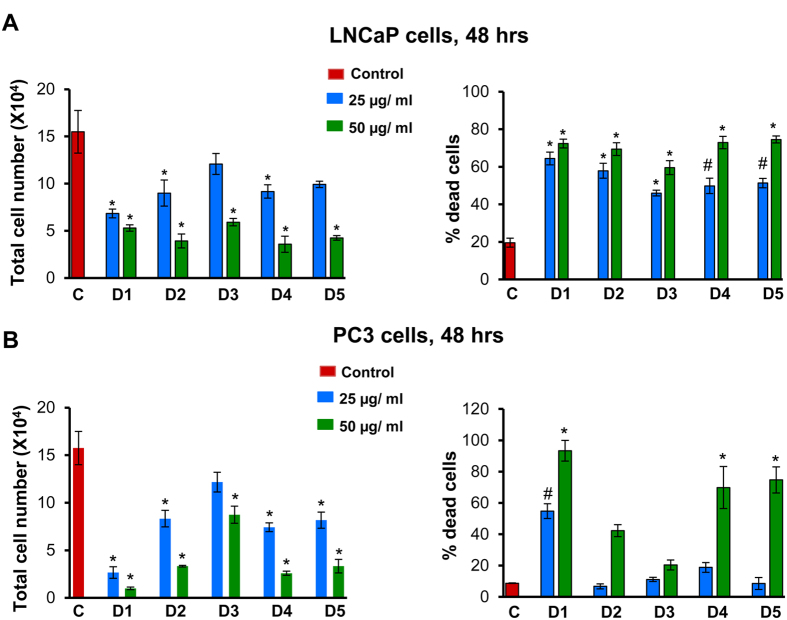
Comparison of various parts of Graviola for cytotoxicity against PCa cells. **(A,B)** LNCaP and PC3 cells were seeded at the density of 5 × 10^4^ cells/well in 6-well plates. After 24 h of seeding, cells were treated with 25 and 50 μg/ml concentrations of Graviola extracts (D1: seeds extract; D2: pulp extract; D3: exocarp extract; D4: leaves extract; D5: twigs extract) for 48 h. At the end of 48 h, cells were harvested and counted as mentioned in ‘Materials and Methods’, and total cell number and percentage of dead cells are presented as mean ± SEM. *p ≤ 0.001; ^#^p ≤ 0.01.

In earlier studies, several commercial preparations of Graviola had been tested for their anti-cancer effects^25^,^26^,^29^. For standardization purposes, we started with various Graviola plant parts (seeds D1, pulp D2, exocarp D3, leaves D4, and twigs D5), prepared their extracts, and compared their cytotoxicity against human PCa cells. LNCaP and PC3 cells were treated with equal amounts (25 and 50 μg/ml) of these extracts (D1–D5) for 48 h, and total and dead cell numbers were determined by trypan blue exclusion assay. As shown in Fig. 2A, all the 5 extracts showed quite similar dose-dependent effect in terms of a decrease in total cell number and an increase in percentage cell death in LNCaP cells. In PC3 cells too, all the extracts showed cytotoxicity, with the seed extract (D1) displaying the highest relative efficacy (Fig. 2B). Overall, seed extract showed highest efficacy against PCa cells. However, Graviola pulp extract (GPE) (D2) was selected for subsequent studies with the rationale that Graviola pulp is the only part that is widely consumed by humans, and therefore, it has the greatest potential for translation to chemoprevention in humans.

### GPE inhibits hypoxia-induced NOX activity in human PCa cells

**Figure 3:**
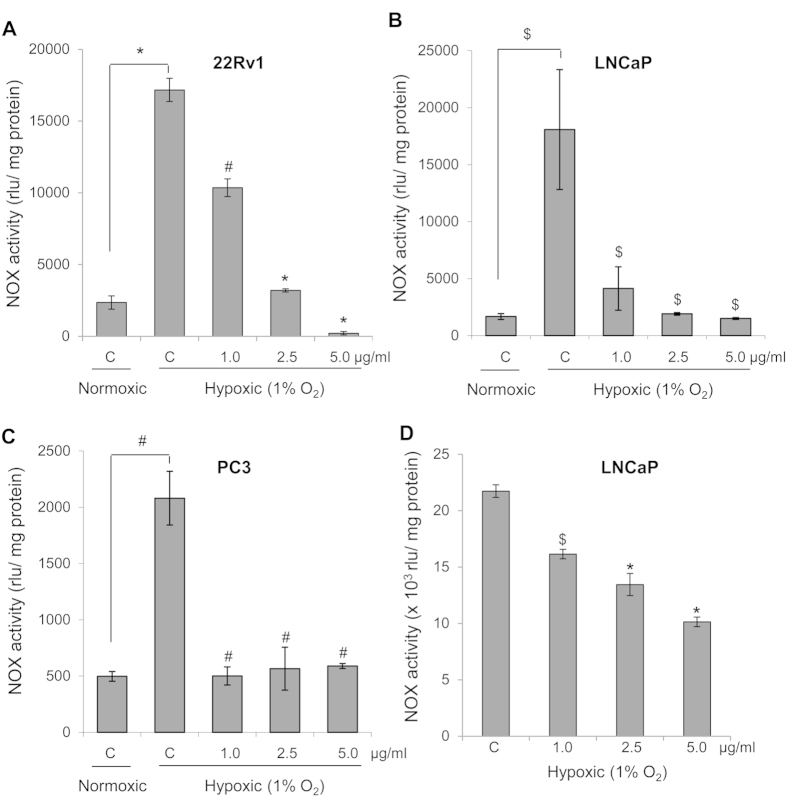
GPE inhibits NOX activity in human PCa cells. **(A–C)** Human PCa 22Rv1, LNCaP and PC3 cells were seeded at the density of 4 × 10^5^ cells/60 mm culture dishes. After 24 h of seeding, cells were treated with 1, 2.5 and 5 μg/ml concentrations of GPE under hypoxic condition (1% O_2_) for 24 h. In each case, cells cultured under normoxic condition (21% O_2_) served as relevant control. At the end of 24 h, cells were harvested and NOX activity was measured as mentioned in ‘Materials and Methods’ and represented as rlu/mg protein. **(D)** Direct inhibition of NOX activity by GPE was assessed by pre-incubating GPE (1.0–5.0 μg/ml) with cellular homogenates and NOX activity was measured as mentioned in ‘Materials and Methods’ and represented as rlu/mg protein. Each value represents mean ± SEM of three samples for each treatment. ^$^*p* ≤ 0.05; ^#^*p* ≤ 0.01; *p ≤ 0.001.

As mentioned above, NOX is a major regulator of PCa growth and progression, so next we determined GPE efficacy to inhibit NOX activity in PCa cells. First, we compared the NOX activity under normoxic (21% O_2_) and hypoxic (1% O_2_) conditions in PCa cells. In all the three PCa cell lines, compared to normoxic condition, NOX activity was induced under hypoxic condition by 7-, 10- and 4- folds in 22Rv1, LNCaP and PC3 cells, respectively (Fig. 3A–C). GPE treatment (1, 2.5 and 5 μg/ml) significantly inhibited the hypoxia-induced NOX activity in 22Rv1 cells by 39–98%, LNCaP cells by 77–91% and in PC3 cells by 71–75% (Fig. 3A–C). Importantly, NOX activity was extremely low in non-neoplastic prostate epithelial PWR-1E cells and was not affected following GPE treatment (1–5 μg/ml; data not shown). In addition, we also assessed the direct effect of GPE on NOX activity by incubating cellular homogenates with GPE (1–5 μg/ml). As shown in Fig. 3D, GPE could also directly inhibit NOX activity in LNCaP cells; and we had similar results in 22Rv1 cells (data not shown).

### GPE decreases the expression of various subunits of NOX system and HIF-1α/ HIF-2α transcription factor in human PCa 22Rv1 cells under normoxic and hypoxic conditions

**Figure 4:**
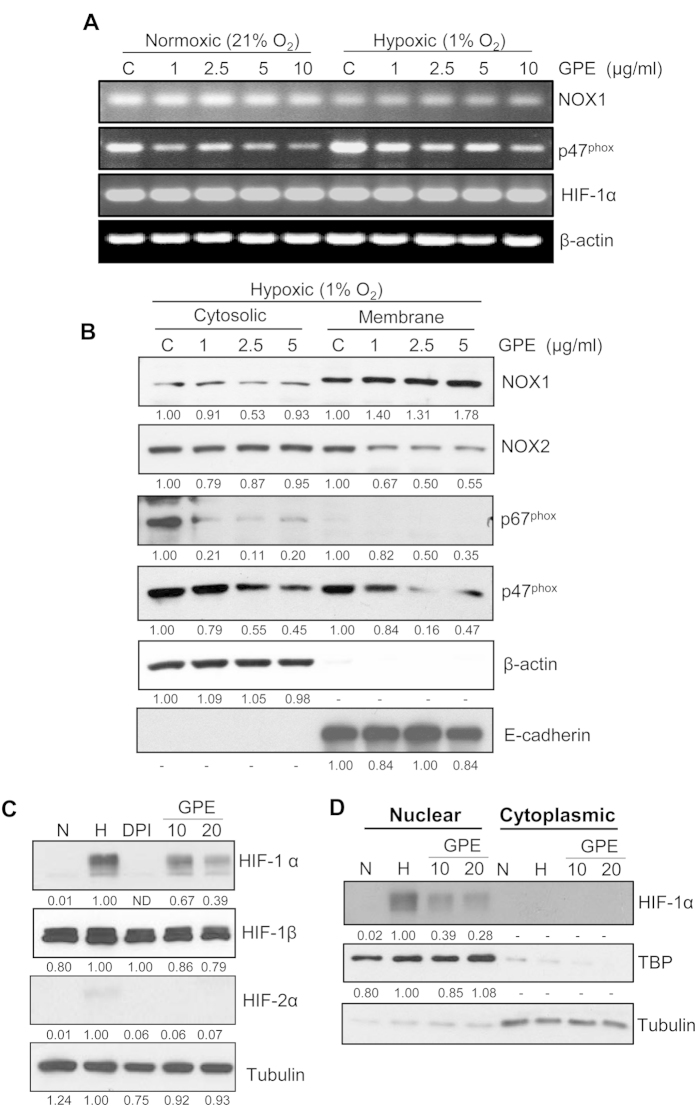
GPE inhibits the expression of members of NOX system and HIF-1α in human PCa 22Rv1 cells. **(A)** 22Rv1 cells were treated with GPE (1–10 μg/ml) under normoxic (21% O_2_) or hypoxic (1% O_2_) conditions for 24 h. Thereafter, cells were collected and analyzed for mRNA expression of NOX1, p47^phox^ and HIF-1α by RT-PCR using gene specific primers as mentioned in ‘Materials and Methods’. β-actin served as an endogenous internal standard. **(B)** 22Rv1 cells were treated with GPE (1–5 μg/ml) under hypoxic (1% O_2_) condition for 24 h. Thereafter, cells were collected and cytosolic and membrane fractions were prepared and analyzed for levels of NOX1, NOX2, p67^phox^ and p47^phox^ by immunoblotting. β-actin and E-cadherin were used to check the purity of cytoplasmic and membrane fractions as well as for loading control. Densitometry data presented below the bands are ‘fold change’ as compared with control (DMSO treated) after normalization with respective loading control. **(C)** 22Rv1 cells were treated with DPI (20 μM) and GPE (10 and 20 μg/ml) under hypoxic (1% O_2_) condition for 6 h. Cells cultured under normoxic condition (21% O_2_) served as relevant control. Thereafter, cells were collected and total cell lysates were prepared and analyzed for HIF-1α, HIF-1β, HIF-2α and tubulin. Densitometry data presented below the bands are ‘fold change’ as compared with hypoxia control (DMSO treated) after normalization with respective loading control. **(D)** 22Rv1 cells were treated with GPE (10 and 20 μg/ml) under hypoxic (1% O_2_) condition for 6 h. Cells cultured under normoxic condition (21% O_2_) served as relevant control. Thereafter, cells were collected and nuclear/cytoplasmic fractions were prepared and analyzed for HIF-1α expression. Membranes were also probed for TBP and tubulin as loading control for nuclear and membrane fractions, respectively. Densitometry data presented below the bands are ‘fold change’ as compared with hypoxia control (DMSO treated) after normalization with respective loading control. Abbreviations: N: Normoxic; H: Hypoxic; GPE: Graviola pulp extract; DPI: Diphenyleneiodonium.

We selected 22Rv1 cells to elucidate the mechanism associated with GPE-mediated inhibition of NOX activity by evaluating the expression of various subunits of NOX system at the mRNA level by semi-quantitative RT-PCR. Results showed that GPE slightly decreased the expression of NOX1 only under hypoxic conditions, but decreased the expression of p47^phox^ under both normoxic and hypoxic conditions (Fig. 4A). Further, we evaluated the mRNA expression of HIF-1α transcription factor in 22Rv1 cells, and the results showed that there was no change in the expression of HIF-1α mRNA levels (Fig. 4A). The literature suggests that the regulatory subunits of the NOX system (p67^phox^ and p47^phox^) are primarily cytosolic that translocate to the cellular membrane following different stimuli (e.g. hypoxia) to activate the catalytic subunits (gp91^phox^). Accordingly, next we assessed the effect of GPE on the cytosolic and membrane level of NOX1, NOX2, p67^phox^ and p47^phox^ under hypoxic conditions. Results showed that there was no significant change in NOX1 and NOX2 level in the cytoplasmic fraction, while only NOX2 level decreased in the membrane fraction following GPE treatment (Fig. 4B). The level of p67^phox^ and p47^phox^ decreased in both the fractions, but a relatively lower amount of p67^phox^ was detected in the membrane fraction (Fig. 4B).

As mentioned above, under hypoxic conditions, NOX and HIF-1α reciprocate and control each other’s expression at various levels. Therefore, we subsequently evaluated the effect of GPE treatment on HIF-1α expression via immunoblotting in 22Rv1 cells. As expected, compared to normoxia, under hypoxia, HIF-1α protein expression was strongly increased, and that was strongly inhibited by GPE treatment (Fig. 4C). To confirm that GPE-mediated NOX inhibition was involved in the decreased HIF-1α expression, we also used the NOX inhibitor diphenyleneiodonium chloride (DPI; 20 μM) in this experiment. As shown in Fig. 4C, DPI treatment completely inhibited the hypoxia-induced HIF-1α expression in 22Rv1 cells. We did not observe any significant change in HIF-1β expression in 22Rv1 under hypoxic conditions as well as following GPE treatment, which is expected as HIF-1β is constitutively expressed in these cells. Compared to HIF-1α, we observed extremely low level of HIF-2α in 22Rv1 cells under hypoxic conditions which was strongly decreased by DPI and GPE treatment (Fig. 4C). Next, we prepared nuclear and cytoplasmic fraction to characterize the effect of GPE treatment on the sub-cellular localization of HIF-1α and HIF-2α. As shown in Fig. 4D, HIF-1α was localized primarily in the nucleus, and its expression was strongly inhibited by GPE treatment. Similarly, HIF-2α expression (extremely low) was only in the nucleus, which was completely inhibited by GPE treatment (data not shown). These results suggest that enhancement in NOX activity is an important event under hypoxic conditions, which directly/indirectly regulates stabilization of HIF-1α expression in PCa cells.

### GPE inhibits the clonogenicity of human PCa cells under normoxic and hypoxic conditions

**Figure 5:**
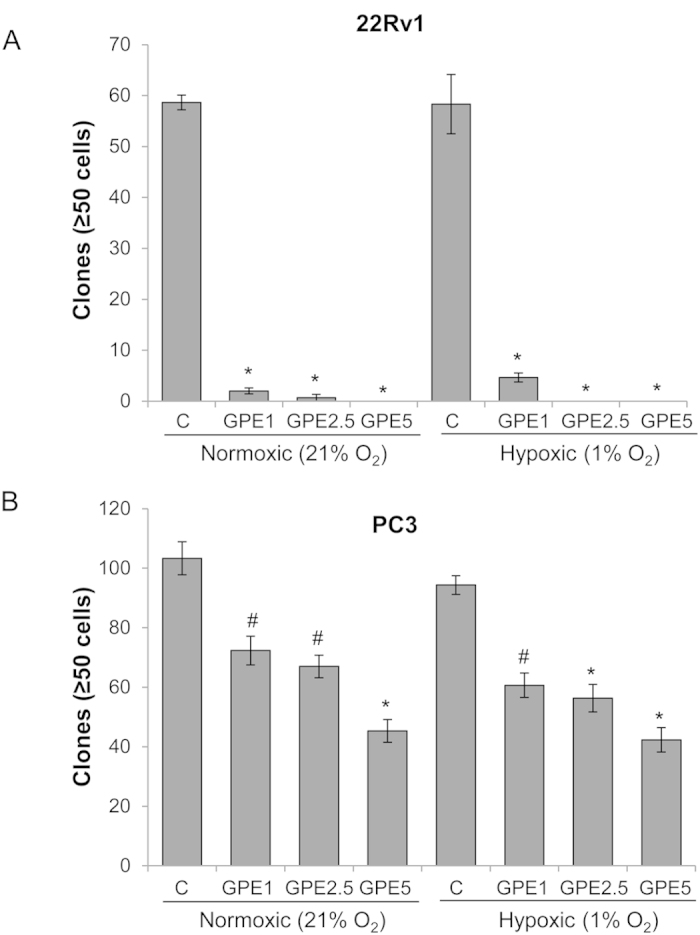
GPE inhibits the clonogenicity of human PCa cells under normoxic and hypoxic conditions. **(A,B)** Human PCa 22Rv1 and PC3 cells (~1 × 10^3^ cells per well) were cultured in 6-well plates and treated with GPE (1–5 μg/ml) under normoxic (21% O_2_) or hypoxic (1% O_2_) conditions for 48 hrs. Thereafter, cells were maintained under normoxic conditions and clones were counted after 7 days. Each value represents mean ± SEM of three samples for each treatment. ^#^*p* ≤ 0.01; *p ≤ 0.001.

Earlier studies have shown that NOX activity inhibition results in a reduction in the survival of cancer cells^2^,^3^,^8^,^30^; therefore, next we determined the effect of GPE treatment on the colony forming potential of PCa cells in the clonogenic assay. As shown in Fig. 5A, GPE treatment (1–5 μg/ml) almost completely inhibited the clone formation by 22Rv1 cells under both normoxic and hypoxic conditions. Similarly, GPE treatment reduced the clonogenic potential of PC3 cells under both normoxic (by 30–56%) and hypoxic (by 35–55%) conditions in a dose-dependent manner (Fig. 5B); however, GPE efficacy was not as prominent in PC3 cells compared with 22Rv1 cells. This could probably be due to the genetic differences between the two cells lines where 22Rv1 cells express androgen receptor (mutated but functional), p53 and PTEN while PC3 cells lack androgen receptor, p53 and PTEN expression.

### GPE specifically reduces cell viability of human PCa cells under normoxic and hypoxic conditions

**Figure 6:**
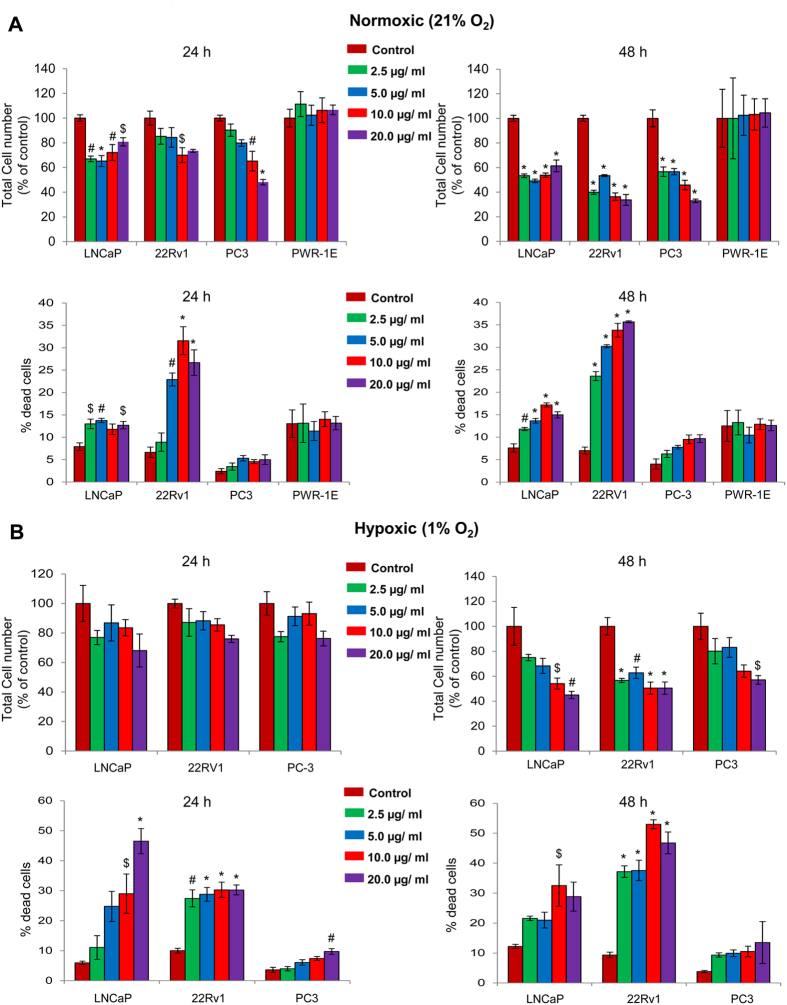
GPE reduces cell viability of human PCa cells under normoxic and hypoxic conditions. **(A,B)** 22Rv1, LNCaP, PC3 and PWR-1E cells were seeded at the density of 5 × 10^4^ cells/well in six well plates. After 24 h of seeding, cells were treated with 2.5–20 μg/ml concentrations of GPE under normoxic (21% O_2_) or hypoxic (1% O_2_) conditions for 24 and 48 h. At the end of each time point, cells were harvested and counted as mentioned in ‘Materials and Methods’, and total cell number and percentage of dead cells are shown. Each value represents mean ± SEM of three samples for each treatment. ^$^*p* ≤ 0.05; ^#^*p* ≤ 0.01; *p ≤ 0.001.

Next, the effect of GPE treatment (2.5, 5, 10 and 20 μg/ml) for 24–48 h was examined in three human PCa cell lines by trypan blue exclusion assay under both normoxic and hypoxic conditions. As shown in Fig. 6A,B, GPE treatment caused a significant decrease in total cell number and an increase in cell death in all the three PCa cell lines under both normoxic and hypoxic conditions. We also assessed the cytotoxicity of GPE treatment on non-neoplastic prostate epithelial PWR-1E cells by trypan blue exclusion assay. As shown in Fig. 6A, no significant change was observed in total cell number as well as percentage cell death following GPE treatment (2.5–20 μg/ml) in PWR-1E cells. These results clearly indicate the specific cytotoxicity of GPE against PCa cells with no cytotoxic effect against non-neoplastic prostate epithelial cells.

### Characterization of GPE

**Figure 7:**
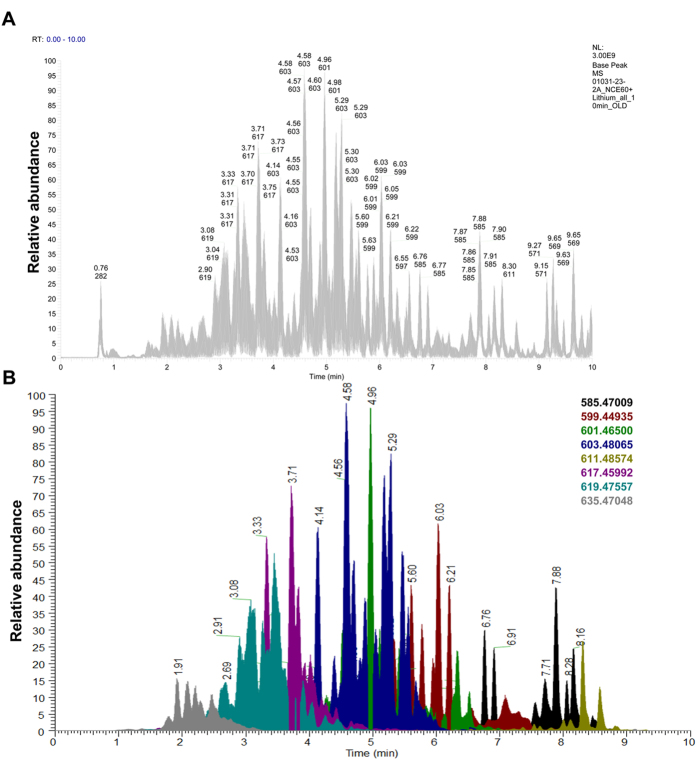
Chemical characterization of GPE. Sample was analyzed as detailed in the methods. **(A)** The total ion chromatogram of the GPE. **(B)** The extracted ion chromatograms, detailed to the right, overlaid with each other. The color coding subdivides the acetogenins based upon their molecular ions.

**Figure 8:**
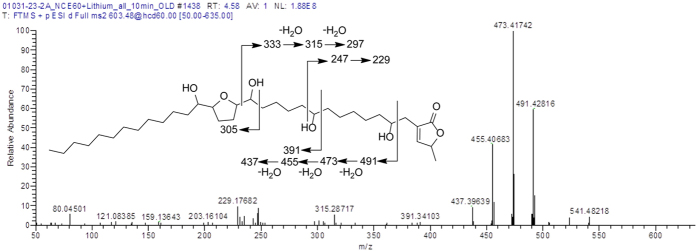
Example characterization of acetogenins. The MS/MS spectrum of the peak at 4.58 min (coded blue in Fig. 7). These data illustrate the common fragmentation patterns observed with acetogenins. In particular, note the fragments adjacent to hydroxy moieties, thereby facilitating the elucidation of the positioning of the THF ring and each hydroxy group. Based on these data and the associated literature, this compound is tentatively identified as annonacin.

In the direction of characterizing the active constituents in GPE, the sample was subjected to ultraperformance liquid chromatography–high resolution tandem mass spectrometry (UPLC-HRMS-MS/MS) (Fig. 7). As reported previously, to increase the sensitivity of the MS/MS fragments for acetogenins, lithium fluoride was infused post-column to form [M+Li]^+^ adducts^31^. Furthermore, higher-energy collision dissociation (HCD) was used, as it affords high resolution fragmentation, thus facilitating the elucidation of the structural backbone. Fragmentation typically occurs on either side of the hydroxy groups, and this pattern assists in identifying the placement of the tetrahydrofuran ring along the hydrocarbon chain in relationship to the lactone ring (Fig. 8) as well as the placement of any ancillary hydroxy groups. These patterns were compared to those reported in literature to tentatively identify the structures of the acetogenins^31^,^32^,^33^.

## Discussion

PCa is the leading cause of cancer-related incidences and 2^nd^ leading cause of cancer-related deaths in the United States. Increasingly, we have succeeded in early diagnosis of the disease localized mostly at the PIN stage; however, this has not translated into reducing PCa morbidity and mortality. Newly diagnosed PCa patient are mostly kept on watchful waiting and monitored for years as the potential challenges with the treatment exceeds the benefits. These PCa patient populations could be ideal for chemopreventive approaches, as such agents are generally non-toxic, orally bioavailable, and have broad efficacy. However, the main challenges in PCa chemoprevention approaches are reliable biomarkers for predicting the course of disease, as well as, the efficacy of the chemopreventive agents.

Accumulating evidences clearly indicates that NOX activity could affect several of the hallmarks of cancer, including genomic instability, growth, survival, angiogenesis, invasion and metastasis^2^,^3^,^5^,^7^,^10^. There are abundant reports suggesting that the NOX system plays an important role in PCa growth and progression^2^,^3^,^4^,^5^,^8^,^30^. In a pioneer study, Arbiser *et al.* showed that ectopic expression of NOX1 in PCa cells promoted their growth, tumorigenicity and angiogenicity^5^. Lim *et al.* reported that NOX1 is overexpressed in a high percentage of human prostate tumors associated with elevated ROS generation^4^. That study also reported that increased NOX protein correlated with enhanced tumor and metastatic potential of PCa cells^4^. Similarly, NOX5 down-regulation caused growth arrest and apoptosis in PCa cells^30^. Another study by Kumar *et al.* clearly established that NOX-mediated oxidative stress determines PCa aggressiveness^2^. Importantly, NOX (1, 2 and 4) expression is significantly up-regulated in rat ventral prostate following castration^6^. In the present study, we identified that NOX1 and p67^phox^ expression is associated with PCa progression in TRAMP mice. Together, these studies provide a strong support towards the development of non-toxic NOX inhibitors in the prevention and treatment of PCa.

Based upon this rationale, several NOX inhibitors (e.g. apocynin, diphenylene iodonium, 4-[2-aminoethyl]-benzensulfonylfluorid, VAS2870, and VAS3947) have been tested for their anti-cancer efficacy^34^. However, most of the existing NOX inhibitors lack specificity, and high concentrations are needed to exert their biological effects^15^,^34^,^35^. In the present study, we identified that Graviola pulp extract (GPE) could effectively inhibit NOX activity in PCa cells at low concentrations (≥1 μg/ml) together with strong reduction in PCa cell proliferation and clonogenicity. This is exciting since Graviola pulp is extensively consumed by humans around the world, and therefore, any confirmed benefits could be easily translated for human use.

The idea of testing Graviola for anticancer effects is primarily rooted in the presence of Annonaceous acetogenins (derivatives of long chain C35–C37 fatty acid biosynthesized via the polyketide pathway) in its various parts (leaves, stem, pulp, seeds etc). Several of these acetogenins exert cytotoxicity via inhibiting mitochondrial complex I that is involved in ATP synthesis^36^. Based upon that, it was rationalized that acetogenins could specifically inhibit growth of cancer cells that usually have a higher demand for ATP compared to normal cells^36^,^37^. In this regard, Torres *et al.* recently showed that Graviola leaf/stem extract decreases ATP content in pancreatic cancer cells; however, that study did not test Graviola effect on normal pancreatic cells^26^. Furthermore, that study showed that Graviola targeted multiple pathways that regulate metabolism, cell cycle, survival and metastasis of pancreatic cancer cells^26^. Graviola treatment also affected the expression of molecules related to hypoxia and glycolysis (HIF-1α, NF-κB, GLUT1, GLUT4, hexokinase II and lactate dehydrogenase-A) in pancreatic cancer cells^26^. In another study, Dai *et al.* reported that Graviola fruit extract selectively inhibits the growth of human breast cancer cells both *in vitro* and *in vivo* via down-regulation of EGFR expression^25^. Importantly, higher expression of NOX subunits have also been reported in several cancers, including those of the pancreas and breast^7^. There is a possibility that the reported efficacy of Graviola extracts against pancreatic and breast cancer is through NOX inhibition.

Our rationale of testing the Graviola pulp extract for NOX activity inhibition was based upon an earlier study, where a specific annonaceous acetogenin, bullatacin, showed the capability to inhibit NOX activity in HeLa and HL-60 cells^38^. We still need to confirm the presence of bullatacin, or more likely, structurally related analogues, to identify acetogenins in GPE that could inhibit NOX activity. Past studies on the chemistry of annonaceous acetogenins impart an obvious starting point for the full characterization of GPE^29^,^39^. Moreover, recent developments in chromatography and mass spectrometry will enable this task to be completed rapidly and in a reproducible manner on a wide array of substrates, from foods to juices to dietary supplements^31^. Such chemical analysis will be helpful in establishing the amount of bioactive acetogenins consumed by humans in the form of dietary supplements, juices or as food. Pharmacology studies will also be needed to correlate those chemical profiles to ascertain the best combination for targeting NOX activity in cancer cells, particularly *in vivo*. Furthermore, we need to examine whether GPE could also target mitochondrial complex I and ATP production in PCa cells, and these effects contribute to its strong anti-PCa efficacy independent of NOX activity inhibition.

Overall, the present study for the first time showed that GPE is a novel NOX inhibitor and possesses strong efficacy against PCa. Since Graviola pulp is widely consumed by humans and is non-toxic, it could be readily developed for chemoprevention use in normal/high risk individuals as well as for intervention in PCa patients.

## Materials and Methods

### Cell culture and reagents

Human prostate carcinoma 22Rv1, LNCaP, PC3 and non-neoplastic immortalized PWR-1E cells were obtained from American Type Culture Collection (Manassas, VA). 22Rv1 and LNCaP cells were cultured in RPMI1640 medium supplemented with 10% fetal bovine serum (FBS), 100 U/ml penicillin G, and 100 μg/ml streptomycin sulfate in a humidified 5% CO_2_ incubator. PC3 cells were cultured in RPMI1640 medium supplemented with 10% heat inactivated FBS, 100 U/ml penicillin G and 100 μg/ml streptomycin sulfate. PWR-1E cells were cultured in keratinocyte serum-free medium containing 50 μg/ml bovine pituitary extract and 5 ng/ml epidermal growth factor; and supplemented with 10% FBS during cell viability assay. Media and other cell culture materials were from Invitrogen Corporation (Gaithersburg, MD). GPE stock solution (100 mg/ml) was prepared in DMSO and stored at −80 °C. An equal amount of DMSO (vehicle) was present in each treatment, including control; DMSO concentration did not exceed 0.1% (v/v) in any treatment. Lucigenin, NADPH, sucrose, sodium phosphate monobasic and dibasic, Tris, EDTA, EGTA, NAC, DPI and VAS2870 were from Sigma-Aldrich (St Louis, MO). Antibodies for HIF-1α, HIF-1β, HIF-2α, E-cadherin, p47^phox^, and tubulin as well as HRP conjugated anti-mouse and anti-rabbit secondary antibodies were from Cell Signaling (Beverly, MA); p67^phox^, NOX1 and TATA-binding protein (TBP) antibodies were from Abcam (Cambridge, MA); and NOX2 antibody was from Boster Biological technology (Fremont, CA). Enhanced chemiluminescence detection system was from GE healthcare (Buckinghamshire, UK).

### Trypan blue exclusion assay

At the end of desired treatment time, cells were collected and total cell number and dead cell number were determined using a hemocytometer after trypan blue staining.

### Clonogenic assay

22Rv1 and PC3 cells (~1 × 10^3^ cells per well) were plated in 6-well plates. Next day, cells were treated with DMSO or GPE (1, 2.5 and 5 μg/ml) followed by incubation under normoxic (21% O_2_) or hypoxic (1% O_2_) conditions for 48 h. Thereafter, cells were maintained under normoxic conditions (21% O_2_) with DMSO or fresh GPE. At the end of the 7^th^ day, cells were washed twice with ice cold PBS, fixed with a mixture of methanol and glacial acetic acid (3:1) for 10 min and then stained with 1% crystal violet in methanol for 15 min followed by washing with deionized water. Colonies with more than 50 cells were scored and counted under the microscope.

### NOX activity assay

Cultured cells were homogenized in lysis buffer (20 mM KH_2_PO_4_ pH 7.0, 1 mM EGTA, 1 mM phenylmethylsulfonyl fluoride, 10 mg/ml aprotinin, and 0.5 mg/ml leupeptin) by using a Dounce homogenizer (100 strokes on ice). Homogenates were centrifuged at 800 g at 4 °C for 10 min to remove the unbroken cells and debris. For determining NOX activity, 100 μl aliquots of homogenates were added to 900 μl of 50 mM phosphate buffer containing 1 mM EGTA, 150 mM sucrose, 5 mM lucigenin, and 100 mM NADPH, pH 7.0. Photon emission was measured in a luminometer (Promega, Madison, WI) every 1 min for 15 min. There was no measurable activity in the absence of NADPH. Protein concentration was determined using the DC Protein assay system (Bio Rad, Hercules, CA). Superoxide anion production was expressed as relative chemiluminescence (light) units (RLU)/mg protein. Direct effect of GPE on hypoxia-induced NOX activity was also analyzed by pre-incubating cellular homogenate for 10 min with 1–5 μg/ml final concentrations of GPE, and NOX activity assay was performed as mentioned above.

### RT-PCR

A semi-quantitative reverse transcriptase–polymerase chain reaction (RT-PCR) analysis of NOX1, p47^phox^ and HIF-1α messenger RNAs (mRNAs) in 22Rv1 cells was performed using gene-specific primers. Total RNA was extracted from DMSO or GPE-treated 22Rv1 cells with Tri reagent (Sigma-Aldrich, St. Louis, MO) following the manufacturer’s instructions. Total RNA was treated with RNase-free DNase (Thermo Scientific, Rockford, IL) and the integrity of RNA was determined by running on agarose gel prior to further downstream application. The RT-PCR was carried out using one-step RT-PCR kit (Qiagen, Valencia, CA) according to the manufacturer’s instructions and total RNA (200 ng) was taken as template for each reaction. β-actin gene was taken as endogenous internal standard. NOX1 oligonucleotide sequences for PCR primers were 5′-GGTTGTTTGGTTAGGGCTGA-3′ and 5′-CTGGAGAGAATGGAGGCAAG-3′. p47^phox^ oligonucleotide sequences for PCR primers were 5′-CCCGATACCCAGTTTCAGTG-3′ and 5′-GGAAGGTCTCCTTGAGGGTC-3′. HIF-1α oligonucleotide sequences for PCR primers were 5′-CAGAGCAGGAAAAGGAGTCA-3′ and 5′-AGTAGCTGCATGATCGTCTG-3′. β-actin oligonucleotide sequences for PCR primers were 5′-TCCTTAATGTCACGCACGATTT-3′ and 5′-GAGCGCGGCTACAGCTT-3′.

### Graviola extracts preparation and GPE chemical characterization

Samples of *Annona muricata* plant parts (i.e. seeds, pulps, exocarp, leaves, and twigs) were acquired from the Philippines via Trish Flaster of Botanical Liaisons (Boulder, CO), and a voucher specimen (NCU633520) was deposited in the herbarium of the University of North Carolina at Chapel Hill. The samples were processed using procedures described in detail previously^40^. Briefly, to each 1000 mL flask containing a sample of ~25 g of powdered plant material (where all parts were treated separately) 240 mL of 1:1 MeOH/CHCl_3_ were added. The samples were stirred overnight at room temperature, filtered using vacuum filtration, and the remaining residues were washed with MeOH. To the filtrate, 360 mL of CHCl_3_ and 600 mL of water were added. The mixtures were stirred for ½ h, and then transferred into separatory funnels. The bottom layers were drawn off into round-bottom flasks, which were evaporated to dryness. The dried organic extracts were re-constituted in 200 mL of 1:1 MeOH/CH_3_CN and 200 mL of hexanes and transferred to separatory funnels. The biphasic solutions were shaken vigorously. The MeOH/CH_3_CN layers were evaporated to dryness under vacuum.

HRESIMS was performed in positive ionization mode on a Thermo Q Exactive Plus mass spectrometer (ThermoFisher, San Jose, CA) equipped with an electrospray ionization source. UPLC was carried out on a Waters Acquity system [using a BEH C18 (2.1 × 100 mm, 1.7 μm) column (Waters Corp., Massachusetts, USA) equilibrated at 40 °C]. A mobile phase consisting of CH_3_CN:H_2_O (both acidified with 0.1% formic acid) at a flow rate of 300 μL/min was used for analysis with lithium fluoride (2 mM in MeOH) infused post-column at a flow rate of 5 μL/min. The gradient started with 70:30 (CH_3_CN:H_2_O) for 1 min then increased linearly to 100% CH_3_CN over 7 min, holding for 1.5 min and then returning to the starting conditions within 0.5 min. An Acquity UPLC photodiode array detector was used to acquire PDA spectra, which were collected from 190–500 nm with 4 nm resolution. Higher-energy collisional dissociation (HCD) was performed using normalized collision energy (NCE) at 60%.

### Immunohistochemistry (IHC)

Paraffin-embedded TRAMP and non-transgenic prostate tissue sections from earlier completed studies were used to determine the expression of NOX1 and p67^phox^ by IHC^27^,^28^. Briefly, sections were incubated with anti-NOX1 or anti-p67^phox^ antibody, followed by a specific biotinylated secondary antibody (1:250 dilution), and then conjugated HRP streptavidin and DAB working solution, and counterstained with hematoxylin. Stained sections were analyzed by Zeiss Axioscope 2 microscope and images captured by AxioCam MrC5 camera at 100x magnification. Immunoreactivity (represented by brown staining) was scored as 0+ (no staining), 1+ (weak staining), 2+ (moderate staining), 3+ (strong staining), 4+ (very strong staining).

### Immunoblotting

Total cell lysates, nuclear and cytoplasmic fractions were prepared following our published protocols^41^,^42^ and cytosolic/membrane fractions were prepared using subcellular protein fractionation kit (Thermo scientific, Rockford, IL) following the manufacturer’s instructions. Approximately, 40–60 μg of protein lysate per sample was denatured in 2× sample buffer and subjected to sodium dodecyl sulfate–polyacrylamide gel electrophoresis (SDS-PAGE) on Tris–glycine gel. The separated proteins were transferred on to nitrocellulose membrane followed by blocking with 5% non-fat milk powder (w/v) in Tris-buffered saline (10 mM Tris–HCl, pH 7.5, 100 mM NaCl, 0.1% Tween 20) for 1 h at room temperature. Membranes were probed for the protein levels of desired molecules using specific primary antibodies followed by the appropriate peroxidase-conjugated secondary antibody and visualized by ECL detection system. To ensure equal protein loading, membranes were stripped and re-probed with appropriate loading control. The bands were scanned with Adobe Photoshop 6.0 (Adobe Systems, San Jose, CA) and band density was determined using the Image J software. In each case, blots were subjected to multiple exposures on the film to make sure that the band density is in the linear range.

### Statistical analysis

Statistical analyses were performed with Sigma Stat software version 2.03 (Jandel scientific, San Rafael, CA) and Prism 5.0 (Graphpad software, La Jolla, CA). One-way ANOVA followed by Tukey’s test was used for multiple comparisons and statistically significant difference was considered at *p* ≤ 0.05.

## Additional Information

**How to cite this article**: Deep, G. *et al.* Graviola inhibits hypoxia-induced NADPH oxidase activity in prostate cancer cells reducing their proliferation and clonogenicity. *Sci. Rep.* **6**, 23135; doi: 10.1038/srep23135 (2016).
